# The Gradual Changes and Their Unknown Cause

**Published:** 1920-06-05

**Authors:** 


### DISEASE AND THE PASSING YEARS.

### The Gradual Changes and their Unknown Cause.

To what extent the many diseases which attack mankind remain true to their original type, forms a subject which has periodically attracted attention from the earliest times, but until recent years the balance of opinion has apparently held that the types had not themselves undergone any real change, whereas the attitude of the physician—or rather the fashionable conception of disease in his particular period—had created a change in the professional mind, and the actual conditions and symptoms of his patients remained substantially the same. In recent years Clifford Allbutt and others have repeatedly instanced the fact that disease has never been a fixed entity, and that all sickness in relation to the human body is subject to the same thousands of subtle influences which control the whole economy of the animal kingdom. Little enough do we still know of these influences, and perhaps it is as well, for if we could truly pigeon-hole medicine as the text-books are often too prone to suggest, its study would lose the wonderful attraction which it must ever possess. No better demonstration of this element of uncertainty has been given than the recent address by Sir H. D. Rolleston at the meeting of the American Medical Association. In a country notorious for its materialism and a general striving to reduce every phase of science to a mathematical precision, this review by a man of wide and recent experience did much both to interest and stimulate a distinguished audience.

### Classical Changes.

In quoting the facts that scarlet fever, though perhaps not less frequent, was less fatal than formerly, and that influenza had certainly undergone an increase in virulence since the type of 1889 had been accepted as a standard, classical instances were provided, and although with the latter there has probably been no disease with so voluminous a recent literature, it is interesting to note the meaning of British statistics, small though they are. There were two waves of influenza in 1918. In the first wave but 4 per cent. of the cases had complications and the mortality was 1.03 per cent. During the second wave, recurring in October 1918, the incidence of complications was 7 per cent. and the mortality 3 per cent. Although this rapid alteration proves on a first count that "disease" is not a clinical entity in the old sense, yet it suggests rather the great question of "virulence."

It appears probable that alterations in clinical features usually regarded as typical of any one disease must be due to one or both of two chief causes, namely (*a*) differences in the virulence of the bacterium concerned, and (*b*) changes in the actual resistance of the patient. It is reasonable to suppose that, parallel with laboratory experiments on animals, a highly virulent bacterium infecting a patient with low resistance will produce a fulminatory type of the disease, whereas an attenuated virus in the same person or in an individual with good resistance will produce a very mild type of the disease or possibly no effect at all. That is the simple explanation which finds easy acceptance, but it is not in truth so certain that changes are not due to other as yet unknown or partially recognised factors. For example, temperature, concentration of carriers and of hosts, and conditions of transference are realised as playing a direct part in the spread of disease, but how exactly do they influence the virulence and infective vitality of the bacteria? A great deal of somewhat irrelevant speculation has resulted in a vague idea that there are cycles of activity for organisms, apart from simple seasonal variations. It would seem there are periods of activity followed by long periods of rest, but how and why do these rests result? Local explanations have been legion, but most are singularly inconclusive.

### Secondary Infection.

One point, however, stands out. In the majority of disease epidemics there has been at first a slow increase in severity among the early cases, and then a marked upward jump in the mortality figure at a date when secondary infection of the system of pus-forming organisms became common. Presumably the general lowering of resistance to bacteria became progressively greater, until a secondary outbreak of activity by pus-forming micrococci is thereby encouraged. This was well illustrated by the marked difference between influenza during the first wave and the same infection after secondary invasions by hæmolytic streptococci and pneumococci became common. Also we have to remember the possibility that *double* infection may occur—infection by two pathogenic organisms entering the system at the same particular moment. The writer has notes of a number of otherwise typical lobar pneumonia cases in young adults where two distinct and definite crises occurred, and the clinical signs and charts could be readily interpreted as meaning a double infection, with the activity and pathogenic period of the two organisms superimposed. A bedside view of these cases in relation to "day of disease," etc., was extraordinarily different from the classical picture of a lobar pneumonia which has been handed on from text-book to text-book, yet the diagnosis, treatment, and general considerations were essentially the same.

### Bacteriological Variants.

Indeed, as one proceeds with such a review, the real cause of change in clinical disease seems to lie more and more in the realms of bacteriology, and in that we have yet so much to learn of this young science it is not surprising that the lecturer found considerable difficulty in bringing his remarks below the plane of interesting generalities. It is well for those who are inclined to think we have reached any

**Disease and the Passing Years**—(*continued*).

degree of finality in attaching a specific microbic cause to even the best-known diseases to remember that there is evidence accumulating daily that we must recognise the possible occurrence of bacteriological variants, species and sub-species, which, owing to the extraordinary number of generations passed through in a brief time by the organisms, can readily give rise one to another. It has been suggested that cerebro-spinal fever, acute poliomyelitis, encephalitis lethargica, and influenza are all variants of the same morbid process. The elaboration of our knowledge of so-called "enteric fever" is another example. The amazing number of strains of pneumococci which immunity studies and attempts at serum treatment have brought to light speak of an immense untried field still ahead of us.

But passing from such likely changes in the organism itself, let us realise that these tiny creatures are probably controlled just as much as ourselves by the laws of species and evolution in relation to external conditions. There is an obvious instance in the effect of general vaccination on reducing the present-day virulence of small-pox as seen in the unvaccinated, another in the gradual but noticeable decline in diphtheria virulence as the use of antitoxin continues year by year. Also local circumstance must play an immense part in controlling what one may call racial resistance, and in both the human and lower animal worlds we have numberless examples of acquired racial immunity to all manner of infecting agents.

Gradual Development.

Probably all such results came from gradual development. Possibly our own local changes in disease are small phases of similar evolution progressing to-day. As Sir Humphry Rolleston cited, we have the instances of devastating measles epidemics among the people of the South Sea Islands, where measles was previously unknown. Even the true-born Colonials visiting this country during the war suffered greatly in this way, for such epidemics have been characterised by an extraordinarily high mortality, and one which is almost unthinkable in connection with measles as we know it at home. Also the same racial immunity may be developing in regard to syphilis. It is said that the virulence of this disease as met with in England is gradually diminishing. May this be true! And also let us hope that, as hygienic knowledge among civilised communities improves, the clinical changes in disease may be towards the development of an increasing racial immunity. Perhaps if the world were always at peace the future might see some approach to this ideal.

